# Development of LXR inverse agonists to treat MAFLD, NASH, and other metabolic diseases

**DOI:** 10.3389/fmed.2023.1102469

**Published:** 2023-02-02

**Authors:** Kristine Griffett, Thomas P. Burris

**Affiliations:** ^1^Department of Anatomy, Physiology and Pharmacology, College of Veterinary Medicine, Auburn University, Auburn, AL, United States; ^2^The University of Florida Genetics Institute, Gainesville, FL, United States

**Keywords:** MAFLD, NAFLD, liver X receptors, dyslipidemia, hypercholesterolemia, cirrhosis, hepatocellular carcinoma, pharmacology

## Abstract

Activation of LXR activity by synthetic agonists has been the focus of many drug discovery efforts with a focus on treatment of dyslipidemia and atherosclerosis. Many agonists have been developed, but all have been hindered due to their ability to efficaciously stimulate *de novo* lipogenesis. Here, we review the development of LXR inverse agonists that were originally optimized for their ability to enable recruitment of corepressors leading to silencing of genes that drive *de novo* lipogenesis. Such compounds have efficacy in animal models of MAFLD, dyslipidemia, and cancer. Several classes of LXR inverse agonists have been identified and one is now in clinical trials for treatment of severe dyslipidemia.

## 1. Introduction

Metabolic-associated fatty liver disease (MAFLD) is a relatively new classification of disease to incorporate the metabolic dysfunction that often occurs within patients presenting with non-alcoholic fatty liver disease (NAFLD) (1). NAFLD has been deemed the world’s leading chronic liver disease leading to liver transplantation or death (2). Currently, there are no universally approved therapies for NAFLD, and its heterogenous pathology often makes it difficult to identify and treat. The incorporation of metabolic dysfunction (e.g., high plasma triglycerides, prediabetes/diabetes, and increased blood pressure) into the definition of NAFLD has a widespread impact on patients and physicians alike and will improve treatment options for those with the disease (1, 3, 4). The current requirements for the diagnosis of MAFLD includes: 1) hepatic steatosis and 2) overweight/obesity, type 2 diabetes mellitus (T2DM), or metabolic dysfunction. Thus, MAFLD diagnosis partly overlaps with NAFLD but is independent of alcohol intake and co-existing causes of liver diseases. MAFLD is considered independent of other liver disease etiologies and allows for the identification of fatty liver in patients displaying other metabolic disorders (2, 3, 5). This review describes some of the processes that contribute to the development of MAFLD, processes that are involved in both MAFLD and NAFLD, and the novelty of targeting the liver X receptor (LXR) pathway using tissue selective inverse agonists to alleviate this disease.

## 2. Pathophysiological processes in the development of MAFLD

The pathophysiology of chronic liver diseases is quite complex, and with the multitude of factors than may contribute to the development of fatty liver, the term MAFLD allows physicians to distinguish between the term “non-alcohol” and be inclusive of key metabolic factors contributing to the disease and potential therapeutic options (6, 7).

### 2.1. Genetic factors involved in MAFLD development

Scientific evidence suggests that genetic factors strongly influence the development of MAFLD, and these factors overlap with those identified as factors for NAFLD and NASH (non-alcoholic steatohepatitis) (1, 5, 8). The patatin-like phospholipase domain-containing protein 3 gene (*PNPLA3*), Membrane bound O-acyltransferase domain containing 7 gene (*MBOAT7*), transmembrane 6 superfamily member 2 gene (*TM6SF2*), and glucokinase regulator gene (*GCKR*) have been the most recognized genes involved in the pathogenesis of fatty liver diseases (9, 10). *PNPLA3* was the first NAFLD-related genetic variant (rs738409; I148M) identified that displays a robust association with the development and severity of NAFLD. This gene is highly expressed in the liver and white adipose tissues and is regulated by insulin signaling via LXR and sterol regulatory binding protein 1c (SREBP1c) pathways (5, 9, 11, 12). Normally, this protein hydrolyzes triglycerides and retinyl esters, however the genetic variant results in impairment of the hydrolase activity, leading to hepatic lipid accumulation.

MBOAT7 functions to remodel phosphatidyl inositol with arachidonic acid and is primarily expressed in the liver (13–15). The rs641738 mutation (C > T), increases the risk of developing not only MAFLD, but an entire spectrum of liver diseases (NASH, cirrhosis, and hepatocellular carcinoma) (16, 17). The TM6SF2 protein facilities hepatic secretion of triglyceride-rich lipoproteins through the VLDL secretion pathway. The variant associated with the development of MAFLD/NAFLD involves a C-to-T substitution at nucleotide 499, which causes a glutamate to lysine change and results in decreased expression of TM6SF2 (18–20). Reduced expression of TM6SF2 results in an increase in hepatic lipid content and is associated with increased hepatic fibrosis in patients.

GCKR regulates glucose influx into hepatocytes to control *de novo* lipogenesis (9). There have been several variants of *GCKR* associated with the development of liver diseases, and the most severe variants lead to overexpression of GCKR, enhancement of hepatic glucose uptake, and increased hepatic lipogenesis (21, 22). Interestingly, this often leads to reduced serum glucose levels (attributed to the enhanced hepatic uptake), and while it may be beneficial by lowering T2DM risk, can alter insulin signaling and further contribute to the progression of MAFLD (16, 23). While these are not the sole genetic factors that contribute to MAFLD and liver disease development, they are currently the most common and well-characterized of the genetic factors. Interestingly, a commonality among these factors is that they either enhance or inhibit pathways involved with insulin and glucose signaling, *de novo* lipogenesis, or lipoprotein secretion and/or packaging.

### 2.2. *De novo* lipogenesis in MAFLD

Because of metabolic dysfunction during MAFLD progression, adiponectin levels are often decreased which leads to the decrease in free fatty acid (FFA) oxidation, which can stimulate *de novo* lipogenesis (DNL) in the liver. DNL is the metabolic pathway that synthesizes saturated fatty acids and monounsaturated fatty acids (MUFAs) from acetyl-coA (5, 19). In patients with MAFLD, the rate of hepatic DNL in greatly increased due to enhanced expression of DNL pathway enzymes that are regulated by the transcription factors sterol regulatory element binding protein-1 (SREBP1) and carbohydrate response element-binding protein (ChREBP) (19). These transcription factors can be activated via glucose flux and insulin signaling, demonstrating how metabolic dysfunction caused by hyperglycemia and/or hyperinsulinemic conditions promotes DNL and steatosis in MAFLD.

Within hepatocytes, FFAs can be esterified to produce TGs, which are either stored as lipid droplets in the liver or packaged as VLDLs into circulation. Because of this, MAFLD patients often present with pro-atherogenic lipid profiles (e.g., low HDL-C and elevated LDL-C, TG, and apolipoprotein B) (11, 19, 22, 24). Humans have a compensatory mechanism to reduce hepatic fat content through the activity of cholesterol ester transfer protein (CETP), which exchanges TG and cholesterol esters between VLDL, HDL, and LDL cholesterol (25–27). However, this mechanism often results in abnormally high HDL cholesterol metabolism and leads to undesirable alterations in lipid profiles in patients. Like NAFLD, dyslipidemia is not constant across the stages of MAFLD (19). Typically, circulating levels of VLDL and LDL are increased in earlier stages, then as MAFLD progresses, patients will develop hepatic fibrosis and circulating levels of apoB-containing lipoproteins decrease (4, 11, 25, 28). Therefore, research indicates that dyslipidemia in MAFLD appears the most pronounced at the earlier stages of the disease.

While DNL contributes to hepatic steatosis, it also is linked to very low-density lipoprotein (VLDL) production via ChREBP activation of microsomal triglyceride transfer protein (*MTTP*) and *TM6SF2*. Several studies have described an increase in VLDL particle size and number due to increased ChREBP and SREBP1 activity, which was observed due to pharmacological activation of the nuclear receptor LXR, a known regulator of DNL (29, 30). Stimulation of the DNL pathway and an increase in the quantity and particle size of VLDL via LXR activation was further confirmed in a study that investigated the stearoyl-CoA desaturase (SCD) enzyme which is involved in the synthesis of MUFAs (29, 30). In MAFLD patients, SCD activity is increased, leading to increased VLDL secretion as well as increased plasma and hepatic triglyceride (TG) levels. Inhibition of SCD, which is a direct target gene of LXR, can suppress hypertriglyceridemia (5, 22).

Other enzymes within the DNL pathway have been identified as targets for alleviating dyslipidemia and MAFLD. The inhibition of fatty acid synthase (FASN), which is the enzyme that synthesizes palmitate from acetyl-CoA and malonyl-CoA, can suppress hepatic steatosis in a variety of mouse models of fatty liver disease (5, 31, 32). While it has yet to be determined whether specifically inhibiting FASN is a valid approach for the treatment of MAFLD, it is also a direct target gene of LXR, suggesting that targeting this nuclear receptor for MAFLD will have beneficial effects in several physiological pathways involved in the pathogenesis of this disease.

### 2.3. Altered lipoprotein processing in the liver attributes to MAFLD

Lipoprotein processing and signaling play an important role in the development of metabolic dysfunction and MAFLD. As mentioned earlier, genetic anomalies and altered DNL processes can contribute to lipoprotein processing defects in MAFLD (5, 16, 19). For example, alterations in VLDL secretion leads to increased lipid content in hepatocytes. However, one area that should be discussed is the role of lipoprotein receptors in the development of MAFLD. The major receptor for cholesterol enriched APOB containing lipoproteins is the LDL receptor (LDLR) (33, 34). Decades of studies have demonstrated that the functional loss of this receptor induces severe hypercholesterolemia and plays a key role in the development of several cardiovascular diseases including atherosclerosis (35–37). *Ldlr* knockout rodents are prone to develop hepatic steatosis particularly when fed a western or high fat diet (38, 39). The role that this receptor plays in MAFLD in humans is unclear, but mutations in the *LDLR* gene are relatively common (40).

*ApoE*-deficient rodents are another model of hypercholesterolemia and cardiovascular disease commonly used to evaluate therapeutics for atherosclerosis (41–43). Like the *Ldlr*-deficient mice, *ApoE*-deficient mice and rats also exhibit steatohepatitis regardless of diet and are likely an important model of MAFLD for drug discovery. APOE in human and mice, affects hepatic lipid balance via VLDL secretion. This altered balance signals for the activation of resident Kupffer cells and infiltration of peripheral macrophages, leading to progression of MAFLD and hepatic fibrosis. As VLDL balance is implicated in the development of MAFLD, there is a role for the VLDL receptor (VLDLR) in this disease as well. VLDLR is typically expressed at low levels in healthy liver and mediates the clearance of triglyceride-rich particles. During MAFLD development, in both mouse models and humans, the expression levels of VLDLR in liver increases enhancing the development of the disease. Recent data demonstrated that in mice lacking the proprotein convertase subtilisin/kexin type 9 (PCSK9) protein have increased expression of VLDLR, LDLR, and fatty acid transporters including CD36, which enhance the development of MAFLD. Loss of *PCSK9* also results in increased hepatic lipid accumulation, impaired beta cell function, and decreased plasma insulin levels.

MAFLD is a highly complex and systemic disease associated with a variety of metabolic changes and has similarities in the development of progression between mouse models and humans. Like NAFLD, MAFLD often begins with the accumulation of lipids in the hepatocytes, driven by a variety of biological factors (e.g., genetic alterations, nutrition, etc.) which is often mediated by VLDL secretion, fatty acid and lipoprotein uptake and processing, and DNL. These altered metabolic signaling processes and downstream effects on insulin signaling/regulation share features with NAFLD, cardiovascular diseases, and T2DM. While there is likely no single therapeutic target that can fully alleviate the complex metabolic dysfunction occurring in MAFLD, the nuclear receptors have proven to be a rich target class for targeting metabolic and cardiovascular diseases.

## 3. Nuclear receptors

Nuclear receptors (NRs) are ligand-regulated transcription factors that orchestrate numerous physiological processes including metabolism, immunity, and development (44). In humans, there are 48 members of the NR superfamily, which include receptors for steroid hormones, retinoic acid, thyroid hormones, fatty acids, and cholesterol metabolites or oxysterols (44–46). Many of the NRs are categorized as orphans since their natural ligands are not yet known. These signaling molecules regulate target gene transcriptional activity through a common mechanism enhanced by their modular structures. NRs have a highly conserved N-terminal DNA-binding domain (DBD) and a C-terminal ligand-binding domain (LBD) connected by a variable (in size and sequence) hinge region. While the LBD is involved in determining ligand specificity, this region also contains a ligand-dependent transactivation function 2 (AF-2) domain, which allows the NRs to recruit co-factors for transcriptional regulation of target genes. These transcriptional co-factors include coactivators that mediate activation of transcription as well as corepressors that mediate silencing of target gene transcription (47–49). While many receptors are considered either exclusively activators (recruit coactivators) or repressors (recruit corepressors) of transcription, several receptors can recruit either coactivators or corepressors depending on the context of a physiological situation.

Several NRs respond to changes in cellular levels of lipids and other metabolic signals including LXRs, farnesoid X receptor (FXR), and peroxisome proliferated-activated receptors (PPARs), and have been identified as therapeutic targets for a variety of metabolic diseases (44, 50). FXR ligands (obeticholic acid, bile acid analogs, etc.) have therapeutic potential for the treatment of NASH with fibrosis (51). PPAR agonists (PPARα and PPARγ) have been clinically used for many years as treatments for diabetes and dyslipidemia while mixed (PPARα/δ/γ) agonists have been evaluated for efficacy against NASH (52–55). Here, we will focus on the LXRs, as they are master regulators of hepatic lipogenesis and are intricately involved in a variety of processes that lead to the development of MAFLD.

### 3.1. Liver X receptors

LXRα and LXRβ were originally identified as orphan members of the NR superfamily (56, 57). Both isoforms form heterodimers with obligate partner Retinoid X Receptor (RXR) and share the conserved domain structure with other NR members including a central DNA-binding domain (DBD) and carboxy-terminal ligand-binding domain (LBD). LXRα is primarily expressed in the liver, kidneys, intestines, and adipose tissues while LXRβ is widely expressed (56, 57). LXRs function as ligand-dependent transcription factors and bind directly to specific DNA sequences known as LXR 4esponse elements (LXREs). Following the discovery of the LXRs in the 1990s, oxysterols were identified as the direct ligands for both receptor proteins (56, 57). Since oxysterols are metabolites of cholesterol and have been shown to be key signaling molecules that indicate sterol levels, it has been elucidated that LXRs function as cholesterol sensors. LXRs can detect relative cholesterol levels through oxysterol metabolites and alter cell physiology as appropriate. LXRs have been shown to regulate cholesterol efflux and transport, as well as regulate lipogenesis and glucose metabolism. Synthetic LXR agonists (T0901317, GW3965) have been shown to display anti-atherogenic properties due to their effects on reverse cholesterol transport mediated by increased cholesterol efflux from peripheral tissues (58–60). However, the activation of LXR by synthetic ligands results in deleterious effects due to increased hepatic lipogenesis and the development of hepatic steatosis (61, 62). This has led to significant difficulties in the development of tissue selective LXR agonists for the treatment of atherosclerosis. The stimulation of hepatic lipogenesis by LXR agonists is due to the increased expression of lipogenic enzymes including FASN, SCD1, and SREBP1c that are direct target genes of LXR (61). LXR expression has been correlated with the degree of hepatic lipid accumulation, as well as hepatic fibrosis and inflammation in patients with liver diseases.

LXRs have a significant role in the regulation of physiological processes involved in the development of MAFLD. As described earlier, numerous physiological pathways can contribute to MAFLD in both mice and humans. This disease is systemic in its development and pathogenesis, beginning with altered lipid storage and metabolism, and progressing in part, due to abnormal metabolic functioning in a variety of tissues and cell types (i.e., T2DM, obesity, inflammation, lipoprotein processing, etc.). Here, we will focus on the physiological processes that LXR regulates, that are distinctively known for enhancing the development and progression of MAFLD.

### 3.2. LXRs are involved in cholesterol and fatty acid metabolism

The role of LXRs functioning as “cholesterol sensors” was confirmed utilizing *Lxr*α-null mice, which accumulated significant amounts of cholesterol in the liver when challenged with a high cholesterol diet due to their inability to activate an LXR-dependent mechanism for excess cholesterol to be converted to bile acids (63). Subsequent studies have identified that LXRs enhance hepatobiliary cholesterol excretion through the direct activation of target genes, *Abcg5* and *Abcg8* (64). Reverse cholesterol transport (RCT) is the process by which excess cholesterol in the periphery is transferred to HDL and transported to the liver for bile acid synthesis and excretion. This process is mediated by the ATP-binding cassette transporter ABCA1 and ABCG1 in macrophages, both of which are direct target genes of LXR (65). Activation of LXRs also induces the expression of several apolipoproteins and genes involved in lipoprotein remodeling including phospholipid transfer protein (PLTP), lipoprotein lipase (LPL), and CETP (66–70). LXR’s role in regulating cholesterol homeostasis via cholesterol transport into and out of the liver has a significant physiological impact on the development of MAFLD. Interestingly, this is not the only component of cholesterol metabolism that is regulated by LXR and has a direct effect on the pathogenesis of MAFLD and other dyslipidemic diseases.

It is well known that high levels of LDL cholesterol (LDL-C) contribute to the development of cardiovascular diseases including atherosclerosis. LDLR is responsible for the uptake of LDL-C and maintenance of systemic cholesterol levels and can be regulated at both the transcriptional and post-transcriptional levels. The sterol regulatory element-binding protein 2 (SREBP2) is the main transcription factor that regulates the expression of LDLR and is activated in response to low cholesterol levels in the cells. LXR however, can control the post-transcriptional regulation of LDLR through its direct target gene, *inducible degrader of LDLR* (*IDOL*) (71–73). The IDOL protein functions as an E3 ubiquitin protein ligase that directly leads to the degradation of LDLR, as well as VLDLR and other related proteins. Studies have shown that treatment with LXR agonists (T0901317 or GW3965) reduces LDLR expression and raises LDL-C plasma levels through an IDOL-dependent mechanism in humans and non-human primates. Genome-wide association studies have identified polymorphisms in the LDLR locus that leads to severe forms of statin-resistant hyperlipidemia (Familial hypercholesterolemia; FH). Patients with FH often are also diagnosed with some form of fatty liver disease.

LXRs are not only important in maintaining cholesterol homeostasis, but they are intricately involved in the regulation of DNL in the liver. Numerous studies have demonstrated the enhancement of fatty acid biosynthesis and VLDL secretion due to LXR agonist treatment. LXR directly controls the transcription of SREBP1c, FASN, and SCD1, and modulates the expression of ChREBP, all of which are directly involved in the pathogenesis of MAFLD and have been discussed earlier.

### 3.3. Synthetic LXR modulators

**FIGURE 1:**
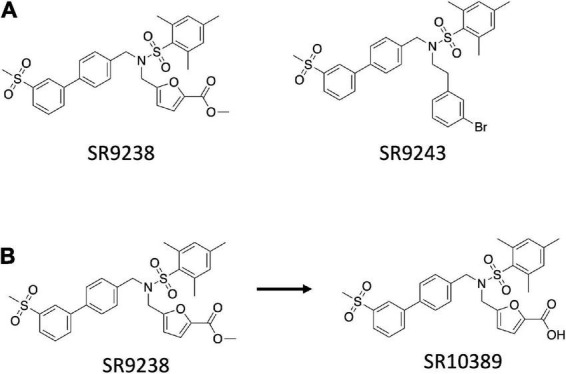
Structure of the SR series of LXR inverse agonists. **(A)** Chemical structure of SR9243 and SR9238. **(B)** Rapid metabolism of the ester functionality of SR9238 to the acid yields a liver specific LXR agonist.

LXRs are master regulators of lipid and cholesterol metabolism and have remarkable anti-inflammatory activities. Because of their multiple roles, they are very interesting drug targets. The major classes of LXR modulators are agonists and antagonists. Agonists bind the LBD of the receptor and recruit coactivator proteins leading to receptor activation and increased expression of downstream target genes. Three LXR agonists are currently in clinical trials for the treatment of atopic dermatitis and advanced solid tumors and lymphoma. LXR antagonists block the binding of agonists and have yet to demonstrate therapeutic utility. A third type of modulator is LXR inverse agonists that was first developed by our group. LXRs have been demonstrated to recruit either coactivators or corepressors depending on the physiological context. We envisioned that development of a LXR ligand that bound to the LBD and selectively enhanced the ability of the receptor to recruit corepressor and suppress the expression of LXR target genes, such as those encoding the DNL enzyme genes, would have the potential to be used in the treatment of metabolic disorders such as MAFLD. The LXR antagonist scaffold (74) was used as an initial point to develop and optimize two novel LXR inverse agonists, SR9238 and SR9243 (Figure 1), that display potent activity for both LXRα and LXRβ and function to very efficaciously recruit corepressor proteins (75–77).

SR9238 exhibits the ability to suppress basal transcriptional activity of LXRα (IC_50_ = 210 nM) and LXRβ (IC_50_ = 40 nM) in a co-transfection assay with a multimerized LXRE luciferase reporter in HEK293T cells (76). In biochemical assays, SR9238 binding to LXRα or LXRβ resulted in recruitment of corepressor NCoR CoRNR box peptides consistent with the ligand functioning as an inverse agonist. Although both NCoR ID1 and ID2 peptides were recruited in a SR9238-dependent manner, there was clear preference for the ID1 peptide for both receptors (76). Treatment of HepG2 cells with SR9238 resulted in significant decreases in the expression of *FASN* and *SREBF1c*, which are key drivers of DNL (76).

Our goal was to develop a LXR inverse agonist that could be used *in vivo* to test our hypothesis that such compounds may hold utility in treated NAFLD. However, a significant concern we had about this type of compound was that if it may decrease RCT even though it may have a beneficial effect on hepatic DNL. Thus, we designed SR9238 with this in mind and SR9238 is a compound with a labile ester group that is rapidly metabolized to a carboxylic acid (Figure 1B). We had noticed previously that certain LXR antagonists would display significant activity with an ester substitution that was lost with hydrolysis of the ester to an acid (78). We observed a similar paradigm with SR9238 as its acid analog (SR10389) was inactive (76). When administered i.p. SR9238 displayed intestinal and hepatic exposure, but no SR9238 was detected in the plasma, skeletal muscle, or brain. Thus, SR9238 provided a tool to assess the ability to target the liver without adversely affecting LXR target genes in peripheral tissues that drive RCT.

When administered to diet induced obese (DIO) mice, SR9238 (i.p.) drove a decrease in expression of *Fasn*, *Srebf1c*, and *Scd1*, which was associated with a significant reduction in hepatic steatosis (76). The reduction in hepatic fat accumulation was accompanied by a decrease in expression of inflammatory genes including *Tnfa* and *Il1b* and a decrease in hepatic F4/80 + cells was also noted. Markers of hepatocellular injury in the plasma (ALP, ALT, and AST) were also significantly reduced consistent with SR9238 improving hepatic function.

The classic DIO mouse provides a model of NAFLD, but hepatic fibrosis (associated with NASH) is not typically noted in this model. However, mice provided a diet high in cholesterol, fructose and trans-fat do develop NASH (79) and we examined the effect of SR9238 in this model as well. This NASH model has been utilized in both C57Bl6 mice and the *ob/ob* leptin deficient mice with similar results, but the disease is accelerated in *ob/ob* mice possibly due to their increased intake of the diet. In the *ob/ob* mice provided this NASH diet, we observed similar effects of SR9238 as we did in the DIO mice with decreased expression of genes encoding DNL enzymes and decreased hepatic steatosis (80). Hepatic weight was decreased, and plasma liver enzymes were also substantially decreased. Importantly, hepatic inflammation was significantly suppressed and hepatic fibrosis decreased by 75% as assessed by collagen staining (80). Although the mechanism of suppression of hepatic fibrosis is not clear, we hypothesized that this was due to the reduction of hepatic steatosis due to suppression of *de novo* lipogenesis leading to reduced inflammation and thus, reduced fibrosis. One interesting point that we noticed in both the NAFLD, and NASH models was that plasma LDL-cholesterol levels (LDL-C) were lowered significantly. In the DIO mice there was a ∼20% decrease whereas in the NASH model there was a ∼50% decrease. We also observed that SR9238 analog, SR9243, that has systemic exposure also displayed similar effects on plasma LDL-C in mice on a normal chow diet (∼50% decrease) (81). At this time, we did not have a proposed mechanism underlying the reduction in LDL-C, but interestingly, a later study of a LXR agonist showed an increase in LDL-C in both non-human primates and in clinical studies (82). This suggested that the LXR inverse agonist mediated decrease in LDL-C we observed in mouse models may be clinically relevant. An independent group assessed the activity of SR9243 in distinct models of NASH including the bile-duct ligation and carbon tetrachloride treatment (83). Huang et al. observed that SR9243 treatment reduced hepatic fibrosis and liver enzymes in both models (83). LDL-C levels were also substantially reduced.

Alcohol consumption is another major driver of liver disease and ethanol also induces hepatic DNL leading to inflammation and fibrosis. Chronic ethanol consumption by mice (Lieber-DiCarli (LD) diet) leads to substantial hepatic steatosis but does not lead to significant hepatic fibrosis. However, addition of “binge” ethanol doses near the end of the chronic ethanol consumption does lead to fibrosis and is a model of alcoholic hepatosteatosis (ASH). We assessed the effects of SR9238 in both models and observed that the drug reduced both fat content in the liver and inflammation (and fibrosis in the ASH model) (84). Like in the NASH model, SR9238 treatment resulted in substantial decrease in the expression of *Srebpf1c* and *Fasn*. Interestingly, treatment also led to an increase in expression of ethanol metabolizing enzymes *Cyp2e1*, *Adh2*, and *Adh3*, suggesting that not only did the LXR inverse agonist suppress DNL but also increased ethanol clearance (84). Given that many patients with steatohepatitis that is driven by both a high fat diet and ethanol consumption, we developed a diet that is composed of both the high cholesterol/trans-fat/fructose and ethanol (WASH diet – western diet and alcohol steatohepatitis). We found that the high cholesterol/trans-fat/fructose diet synergistically acted with the ethanol to enhance hepatic steatosis, inflammation, and fibrosis (85). Importantly, SR9238 treatment was able to suppress the severity of the effects on the liver (85).

As indicated above, one interesting observation we had made with any of the LXR inverse agonists we had tested *in vivo* was that there was a significant decrease in LDL-C. When examining the expression of intestinal genes that changed with SR9238 or SR9243 treatment (i.p.) we found that sterol O-acyltransferase 2 (*Soat2*) gene expression was suppressed by ∼95% (86). This intrigued us given that SOAT2 has been a target for development of drugs to treat hypercholesterolemia and atherosclerosis (87). SOAT2 is an enzyme that converts cholesterol to cholesterol esters and drives intestinal cholesterol absorption (88). Mice with an intestine specific KO of *Soat2* are resistant to development of elevated plasma LDL-C on a high cholesterol diet (89) suggesting that targeting intestinal SOAT2 function or expression may be sufficient to provide this benefit. With our knowledge that SR9243 had no significant oral bioavailability we treated *Ldlr* null mice on a high cholesterol diet and observed that even though there was no liver or plasma exposure when SR9243 was administered orally, LDL-C was substantially decreased and was associated with repression of intestinal *Soat2* expression and increased fecal cholesterol elimination (86). These data provided us with a clear mechanism that was driving the reduction in plasma LDL-C that we consistently observed as well as suggested that such compounds may hold utility in treatment of hypercholesterolemia, particularly in individuals that have mutations in the LDL receptor driving familial hypercholesterolemia.

**FIGURE 2:**
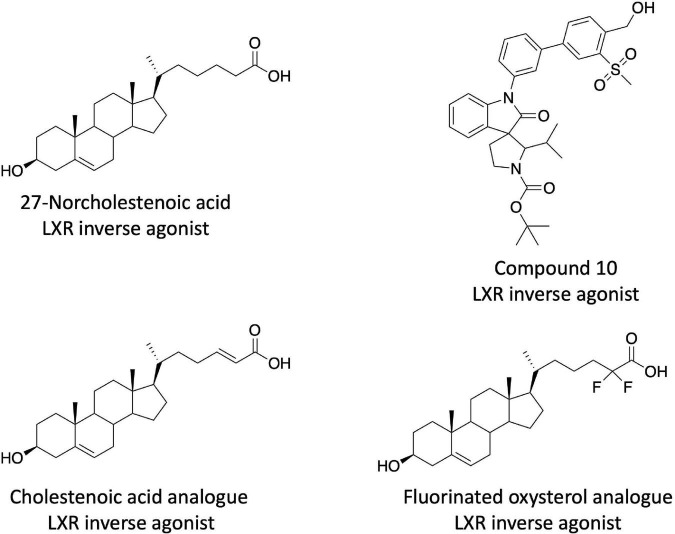
Examples of additional steroidal and non-steroidal LXR inverse agonists.

After the description of the SR9238/SR9243 series of LXR inverse agonists additional chemical scaffolds with similar pharmacological profiles have been described. Burton et al. discovered that several cholestenoic acid analogs displayed LXR inverse agonist activity (Figure 2) (90, 91). These compounds showed the ability to suppress basal transcription in LXR cotransfection assays as well as suppress the expression of LXR target genes (*Fasn*, *Srebf1c*, and *Abcg1*) in HepG2 cells. These compounds drove the recruitment of corepressor proteins to the LXRs, but they did not appear to be very potent as doses greater than 1 μM were required for activity) (90, 91). This group also identified certain fluorinated oxysterol agonists as LXR inverse agonists based on their activity in LXR cotransfection assays in HEK293 cells, but these were also relatively low potency (Figure 2) (92). Chen et al. identified several non-steroidal LXR inverse agonists based on a screen of a compound library that was designed based on co-crystal structures of LXRβ in complex with spiro[pyrrolidine-3,3′-oxindole] agonists (93). These compounds displayed a significant degree of LXRβ selectivity (as much as 100-fold) and the most potent compound was approximately 3.5-fold less potent than SR9238 in a LXRβ cotransfection assay (93). Their most potent compound, 10rr (Figure 2), effectively suppressed *SREBF1c*, *ACC*, *FASN*, and *SCD1* expression in both 3T3-LI adipocytes and HepG2 cells. Compound 10rr suppressed DNL in HepG2 cells consistent with the effects on gene expression and suppressed hyperlipidemia in the Triton WR-1339 induced mouse model (93). Working from the SR9238/SR9243 scaffold, Phenex Pharmaceuticals, developed additional LXR inverse agonists based on a published patent application (94). This intellectual property was licensed by Orsobio, Inc., and a compound (TLC-2716) is currently in phase I clinical trials for treatment of severe dyslipidemia (ClinicalTrials.gov NCT05483998). The structure of TLC-2,716 is not directly disclosed.

## 4. Conclusions and perspectives

LXRs have long been the focus of synthetic ligand development for the purpose of treating dyslipidemia and atherosclerosis. However, this focus has almost entirely been on the development of LXR agonists attempting to drive RCT. It was rapidly determined that such compounds had limiting on-target toxicity associated stimulating DNL resulting in hepatic steatosis and hypertriglyceridemia. Work from our lab focused on utilizing this observed side effect to develop and characterize LXR inverse agonists that actively silence LXR target genes, particularly those that drive *de novo* lipogenesis. With these compounds in hand we were able to demonstrate that were effective in treatment of NASH, ASH, hypercholesterolemia, and cancer in animal models. Several LXR inverse agonist chemical scaffolds have now been identified that display similar pharmacology and even one has entered phase I clinical trials for treatment of severe dyslipidemia.

## Author contributions

KG and TB wrote and edited the manuscript. Both authors contributed to the article and approved the submitted version.
